# Variable alterations in frequencies of CRISPR/Cas9-induced nucleotide deletion and insertion in transgenic maize callus tissues exposed to chromatin modifiers

**DOI:** 10.3389/fgeed.2026.1924094

**Published:** 2026-10-07

**Authors:** Bettina Nagy, Aysha Jameel, Gábor V. Horváth, Katalin Török, Ferhan Ayaydin, Nikolett László, Divya Teja Dondapati, Sándor Mórocz, Zoltán Zombori, Györgyi Ferenc, Dénes Dudits

**Affiliations:** 1 Agribiotechnology and Precision Breeding for Food Security National Laboratory, Institute of Plant Biology, HUN-REN Biological Research Centre, Szeged, Hungary; 2 Functional Cell Biology and Immunology Advanced Core Facility (FCBI-ACF), Hungarian Centre of Excellence for Molecular Medicine (HCEMM), Szeged, Hungary; 3 Faculty of Medicine, Albert Szent-Györgyi Health Centre, Interdisciplinary R&D and Innovation Centre of Excellence, University of Szeged, Szeged, Hungary; 4 Doctoral School of Biology, University of Szeged, Szeged, Hungary; 5 Cereal Research Non-profit Ltd., Szeged, Hungary

**Keywords:** 5-azacytidine, CRISPR/Cas9, gene editing efficiency, heat treatment, maize, nicotinamide, sodium butyrate

## Abstract

Given the growing emphasis on the efficient application of CRISPR/Cas9 in plant genome editing, there is a need for methodologies that enhance targeted mutagenesis efficiency. In the present study, we utilized stable transgenic maize callus tissues harboring active CRISPR/Cas9 vector systems targeting either the *abnormal cytokinin response 1* (*ARE1*) gene or the *potassium channel* gene (*KAT2*). The presented DNA sequencing data indicate that the *KAT2*-targeting vector, which utilizes a 3′-end truncated guide RNA, retained mutagenic activity, albeit at a notably low frequency. We quantified mutagenic events following exposure to elevated temperatures (37 °C for 48 h) and various chromatin-modifying compounds, including sodium butyrate (NaBt), nicotinamide (NA), and 5-azacytidine (AZA). High-temperature treatment alone increased the frequency of nucleotide deletions (5.8-fold relative to the untreated samples) in the *ARE1* target gene. Conversely, in the *KAT2* gene, both deletions and insertions were stimulated (6.4 and 6.2-fold, respectively). As a novel approach, we evaluated the mutagenic efficacy of chromatin-modifying compounds administered in conjunction with heat treatment. The combined application of NaBt and AZA with elevated temperature was particularly effective in increasing insertion frequencies; in the *ARE1* (with NaBt + heat: 17.3-fold) and *KAT2* (with AZA + heat: 21.4-fold) genes. Cell cycle analysis of suspension-cultured cells revealed that chromatin-modifying treatments altered cell cycle progression, evidenced by an accumulation of cells in the G2 phase and a significant reduction in the number of mitotic cells. Subsequent DNA sequencing demonstrated a differential increase in the induction of deletions and insertions, alongside an expansion of the deleted regions.

## 1 Introduction

Although CRISPR/Cas9-mediated gene editing has gained significant applications in plant sciences, different experimental systems still face limitations, particularly regarding the low efficiency of targeted mutagenesis. Mutation frequency depends on both the activity of the Cas9 protein and the chromatin architecture of the recipient plant cells. Chromatin status influences editing efficiency, as a relaxed chromatin structure facilitates Cas9 binding and subsequent DNA repair pathways. The Cas9 RNA-guided endonuclease induces DNA double-strand breaks (DSBs) through its two nuclease domains, RuvC and HNH. DSBs can be repaired via endogenous repair pathways such as canonical non-homologous end joining (cNHEJ), microhomology-mediated end joining (MMEJ), or homologous recombination (HR) (Vu et al., 2024). During the repair process, insertions and deletions (indels) may be generated, disrupting the gene structure at the target site. Different DNA repair pathways can yield characteristic but partially overlapping mutational signatures. Canonical NHEJ often produces small insertions and deletions, whereas MMEJ involves the use of flanking microhomologies and is frequently associated with microhomology-flanked deletions (Salomon and Puchta, 1998; Tan et al., 2020). Previously, Vu et al. (2020) reported high-efficiency, cNHEJ-mediated CRISPR/Cas9-based precise gene insertion by employing a single cut at genomic sites in rice.

Chromatin architecture is tightly linked to the various phases of the cell cycle (Ma, Kanakousaki and Buttitta, 2015).

In addition, repair mechanisms are also cell cycle-dependent; whereas NHEJ is active throughout all cell cycle stages, DSBs persisting through the S and G2 phases until mitosis can be repaired via the MMEJ pathway (reviewed by Sfeir et al. (2024); Přibylová and Fischer (2024)).

At the cellular level, chromatin accessibility, DNA methylation, and histone modifications significantly modulate Cas protein binding and editing efficiency, as heterochromatic (condensed) regions are less accessible to the Cas9-gRNA complex (reviewed by Wei et al. (2025)). Histone deacetylase (HDAC) inhibitors have emerged as powerful tools to transiently open chromatin. By inhibiting HDAC activity and increasing histone acetylation levels, they promote a more relaxed, open chromatin structure, which is generally associated with enhanced DNA accessibility and transcriptional activation. Björnson et al. (2023) demonstrated that the histone deacetylase inhibitors valproic acid (VPA) and sodium butyrate (NaBt) could improve CRISPR cutting efficiency in myelogenous leukemia K562 cells.

Nicotinamide (NA) can directly alter chromatin structure by inhibiting NAD + -dependent enzymes, such as sirtuins—a unique family of Class III histone deacetylases (HDACs) (Imai et al., 2000; Vaquero et al., 2004) and poly (ADP-ribose) polymerases (PARPs). This inhibition prevents the removal of acetyl groups from histones, maintaining chromatin in a relaxed, active state. The influence of chromatin features on CRISPR–Cas9 mutagenesis and DNA repair outcomes has also been demonstrated in plants. Specific chromatin features, such as H3K4me1, H3.3, and H3.1, have been associated with significant variation in CRISPR–Cas9 mutation profiles mediated by the non-homologous end joining repair pathway (Weiss et al., 2022). Previously, we reported that treating maize suspension cultures with NaBt or NA induces chromatin relaxation and improves the efficiency of oligonucleotide-directed mutagenesis, directly linking this effect to HDAC inhibition and chromatin decondensation (Tiricz et al., 2018).

An alternative mechanism of chromatin structural modification relies on DNA methylation: high levels of methylation are generally associated with a closed, transcriptionally inactive state, while reduced methylation can lead to chromatin relaxation and increased transcriptional activity (Hashimshony et al., 2003; Saze et al., 2012; Zhang et al., 2018; Zhong et al., 2021). Methyltransferase inhibitors, particularly 5-azacytidine (AZA) and related cytosine analogs, are widely used to decrease DNA methylation throughout the genome or at specific loci. They have been shown to remodel methylation patterns, alter the expression of stress-responsive and developmental genes, and promote chromatin decondensation in maize and other plants (Yang et al., 2010; Solís et al., 2015).

The Cas9 nuclease function is highly sensitive to temperature; *Streptococcus pyogenes* Cas9 (SpCas9) functions optimally at 37 °C, although it remains active at lower temperatures (Banakar et al., 2021). Given this characteristic, we investigated the effect of varying temperatures on mutation frequency. Kurokawa et al. (2021) also reported that the cleavage activity of SpCas9 is higher at 37 °C than at 22 °C.

In the present study, we utilized stably transformed maize callus tissues carrying a functional full-length guide RNA for the *abnormal cytokinin response 1* (*ARE1*) gene and a truncated guide RNA for the *potassium channel* (*KAT2*) gene. Both guide RNAs were expressed under the control of the rice snoRNA U3 promoter, while Cas9 was expressed driven by the maize ubiquitin (UBI) promoter. This genetic background ensures the continuous induction of mutations in target genes, allowing these calli to serve as an effective test system for monitoring the frequency of mutational events.

A novel aspect of the present study is the monitoring of editing efficiency in maize cells exposed to elevated temperatures, chromatin-modifying compounds, and their combined effects. Heat stress is known to alter chromatin structure in plants; for instance, Korotko et al. (2021) reported a reduction in DNA methylation levels several hours after subjecting *Arabidopsis* plants to heat stress (42 °C) for 24 h. Because the chromatin landscape is dependent on cell cycle phases (Trouth et al., 2025) we analyzed the impact of chromatin-modifying treatments on cell cycle progression. As previously discussed, cell cycle events can dictate the choice of DNA repair pathways, which in turn yield different mutation profiles; therefore, we assessed deletions and insertions independently.

## 2 Materials and methods

### 2.1 Maize transformation

*Zea mays L.* callus cells (SZ17 line), provided by Sándor Mórocz (Cereal Research Institute, Szeged, Hungary), were cultured in the presence of 1 mg/L 2,4-dichlorophenoxyacetic acid (2,4-D; Duchefa) as described by Mórocz et al. (1990). The pRGEB32 plasmid (Addgene, #63142) containing *ARE1* or *KAT2* specific gRNAs was used to generate transgenic maize lines. The gRNAs were previously designed using Benchling® software. The gRNA sequence targeting the *ARE1* gene was GGA​AGA​CCT​TCT​TCT​CGG​AC, and for the *KAT2* gene, it was CAT​TTC​TGA​GAC​ATC​TCT​CA.

Transgenic maize cells were produced *via Agrobacterium tumefaciens*-mediated transformation using the AGL1 *strain*. Maize transformation was performed using culture media 1 or 2 according to the methods of Frame et al. (2006) and Vega et al. (2008), respectively, with minor modifications as described by Souza et al. (2017).

The calli were transferred to glass Petri plates (9 cm) and finely chopped with a sterile surgical blade in ‘infection medium 1’ (Souza et al., 2017) supplemented with 100 µM acetosyringone (Sigma-Aldrich) and 1.5 mg/L 2,4-D. The tissue was washed twice with the same medium. Following the removal of the final wash, 40 mL of the diluted Agrobacterium suspension (prepared in ‘infection medium 1’ and adjusted to an OD_550_ of 0.3–0.4) was added to 50 mL Erlenmeyer flasks containing the calli. To enhance transformation efficiency, a 5-min vacuum infiltration step was applied, followed by a 1-h inoculation at room temperature on a rotary shaker at 100 rpm. The bacterial suspension was then gently blotted on filter paper, and the calli were transferred to ‘co-culture medium 2’ containing 100 µM acetosyringone and 2 mg/L 2,4-D, and incubated at 24 °C for 3 days in the dark.

Subsequently, the calli were washed for 1 h in a liquid co-culturing medium supplemented with 1 g/L Augmentin (Sandoz) and 150 mg/L Timentin (Duchefa) to eliminate Agrobacterium. The calli were then transferred to a resting medium and maintained at 24 °C in the dark for 2 weeks. The resting medium contained 2 mg/L 2,4-D, 0.85 mg/L AgNO_3_ (Sigma-Aldrich), 100 mg/L Augmentin, and 150 mg/L Timentin. Selection was initiated by transferring all calli to a resting medium supplemented with 40 mg/L Hygromycin-B (Duchefa); this step was repeated twice at 2-week intervals. The hygromycin-resistant calli were subsequently placed on a hormone-free maturation medium and incubated for 2–4 weeks at 26 °C.

Transgenic callus lines were tested for the integration of the gRNA cassette, Cas9, and HPT (hygromycin phosphotransferase) genes. Genomic DNA from the transformant calli was isolated using a CTAB-based extraction method according to Doyle and Doyle (1990). For PCR amplification, the following primers were used: gRNA_FWD: 5′- GGC​GTC​TTC​TAC​TGG​TGC​TA -3′; gRNA_REV: 5′-CCC​GGC​CCA​AAT​TGA​AAA​GA -3′; Cas_FWD: 5′-AAA​GAC​CGA​GGT​GCA​GAC​AG-3′; Cas_REV: 5′-ACT​CGC​TGA​TCT​GCT​CGA​TG-3′; HPT_FWD: 5′-CCT​GAA​CTC​ACC​GCG​AC-3′; HPT_REV: 5′-GCT​CAT​CGA​GAG​CCT​GC-3′. The transgenic cell suspension culture was initiated from callus cells grown in liquid medium and cultured in the presence of 1 mg/L 2,4-D as described by Mórocz et al. (1990).

### 2.2 Treatment of transgenic maize cells with heat and chromatin-modifying compounds

Transgenic calli grown on the surface of Gelrite (Duchefa)-solidified N6M medium were treated with histone deacetylase inhibitors, specifically 10 mM NaBt (Sigma-Aldrich) or 5 mM NA (Sigma-Aldrich). These concentrations were selected based on our previous results (Tiricz et al., 2018). Additionally, a DNA methyltransferase inhibitor, 10 μM AZA (Sigma-Aldrich), was utilized as an epigenetic modifier. Cells were treated for 48 h with NaBt or NA, or for 72 h with AZA, under light conditions at 26 °C. Heat treatments were performed separately at 37 °C for 48 h in an incubator.

### 2.3 Flow cytometry

In an attempt to provide information on the alteration of cell cycle events, we isolated nuclei from suspension culture cells derived from the transgenic callus culture and exposed to the above treatments. This change in experimental material was required because callus tissues could not be used for high-quality nuclei isolation. To evaluate DNA content, fixed nuclei suspended in phosphate buffered saline (PBS, pH 7.4) were stained with DAPI (4′,6-diamidino-2-phenylindole (DAPI, AppliChem, Biochemica, Darmstadt, Germany) at a final concentration of 1 μg/mL and incubated on ice for 3 min in the dark. The samples were analyzed using a BD Aria Fusion FACS (BD Biosciences, CA, United States of America), and 10,000 singlet events were acquired per sample using the BD FACSDiva software. For cell cycle analysis, nuclei were first gated based on size and granularity using forward and side scatter parameters. Singlets were then selected using DAPI area versus height discrimination to exclude the doublets. Subsequently, the singlet population was displayed as a histogram of the DAPI fluorescence intensity, and nuclei corresponding to the G1, S, and G2/M phases were identified and quantified using FlowJo analysis software (FlowJo, LLC, Ashland, OR, United States of America). The standard deviation (SD) was calculated from six replicates originating from two different flasks. Statistical analysis was performed with GraphPad Prism 11.0.0 software (GraphPad Software, San Diego, CA, United States of America). For comparisons between multiple groups, including biological replicates, one-way analysis of variance (ANOVA) was performed with Tukey’s honestly significant difference (Tukey’s HSD) as a *post hoc* test to determine pairwise differences between treatments. Differences were considered statistically significant at p < 0.05.

### 2.4 Confocal laser scanning microscopy

Control and treated cells were washed with PBS and fixed with 4% paraformaldehyde in PBS for 40 min on a rocking platform. Cells were washed 3 times for 15 min with PBS, stained with 0.5 μg/mL DAPI in PBS for 15 min and mounted on glass slides with coverslips using Fluromount-G antifade mounting solution (Southern Biotech, AL, United States of America). Images of DAPI stained cells were acquired using Leica DMI8–Stellaris 5 laser scanning microscope (Leica Microsystems Heidelberg, Germany) using 405 nm excitation laser with an emission filter set to 410–435 nm. For mitotic cell count, multiple optical sections were captured using an HC PL APO CS2 20x dry (Numerical aperture, N.A. 0.75) objective to encompass full z-depth of each cell cluster from at least 5 different slide regions. Leica Application Suite X (LAS X, 4.9.0.30221) software was used to count mitotic and/or total cells in the captured images. For high resolution closeup images of representative mitotic cells, an HC PL APO CS2 63x (N.A. 1.35) oil immersion objective was used to acquire extended depth of field z-projections via Leica LAS X software.

### 2.5 Detection of CRISPR/Cas9 gene-specific editing events with next-generation sequencing (NGS)

For the analysis of CRISPR-mediated nucleotide editing, PCR amplifications were performed using the following gene-specific primers containing Illumina adapter sequences:

*ARE1*_Fw: 5′–GTC​TCG​TGG​GCT​CGG​AGA​TGT​GTA​TAA​GAG​ACA​G GTA​ACA​GGA​GTA​GGA​GGG​CG–3′

*ARE1*_Rev: 5′–TCG​TCG​GCA​GCG​TCA​GAT​GTG​TAT​AAG​AGA​CAG AATCGCTTCGGCCTTTCT –3′

*KAT2*_Fw: 5′–GTC​TCG​TGG​GCT​CGG​AGA​TGT​GTA​TAA​GAG​ACA​G GCC​TTA​TCA​GAA​CCT​GGG​AG–3′

*KAT2*_Rev: 5′–TCG​TCG​GCA​GCG​TCA​GAT​GTG​TAT​AAG​AGA​CAG GCT​GCA​ATC​CTT​TTC​GGA​TT–3′

Amplification was conducted using Phusion™ Plus High-Fidelity DNA Polymerase (Thermo Scientific) under the following cycling conditions: an initial denaturation at 98 °C for 3 min; 30 cycles of denaturation at 98 °C for 30 s, annealing at 60 °C for 45 s, and extension at 72 °C for 20 s; followed by a final extension at 72 °C for 10 min. The resulting amplicons of 242 base pairs (bp) for the *ARE1* gene and 223 bp for the *KAT2* gene were utilized for next-generation sequencing (NGS). Prior to sequencing, the PCR products from three independent biological replicates per treatment were pooled.

### 2.6 Index PCR wetlab procedures

The amplicon PCR product concentrations of the amplicon were measured using the Equalbit 1x dsDNA HS Assay Kit (Vazyme) with the Infinite Pro 200 F Nano + Fluorescent Plate Reader (Tecan). Polymerase chain reactions (PCR) and DNA purifications were performed according to the Illumina demonstrated protocol (Illumina, 2013) with the modification that 10 ng of the tagged amplicon template served as the template in index PCR reactions. The products were purified with 1 volume of KAPA Pure Bead (Roche), quantified and their size distribution was determined by capillary electrophoresis on a LabChip GX Touch HT nucleic acid analyzer on the X-Mark HT chip using the DNA NGS 3 k Assay kit (Revvity). The pooled libraries were diluted to 700 pM for 2x150 bp paired-end sequencing with a 300-cycle 10 B or 25 B reagent kit on the NovaSeq X Plus sequencing system (Illumina) according to the manufacturer’s protocol.

### 2.7 Bioinformatic analyzes

Raw sequenced reads, >0.2 M per sample, were demultiplexed and adapter-trimmed using NovaSeq Control Software, while FastQ Toolkit (Illumina) was applied to bin reads having a mean quality score of less than 30 and shorter than 50 bp. The BWA software package was applied to map the filtered sequences to the reference sequences. The raw sequence data was analyzed using Integrative Genomics Viewer (IGV) software. Indels were analyzed within a window spanning from −17 to +6 bp relative to the Cas9 cleavage site. For each nucleotide position, the number of reads carrying a deletion (position PAM–4 for *ARE1* or PAM–6 for *KAT2*) or an insertion (position PAM–3 for *ARE1* or PAM–4 for *KAT2*) was recorded directly from the Integrative Genomics Viewer (IGV) alignment, alongside the total read count at the respective position. To distinguish Cas9-induced mutations from sequencing background noise, the untreated callus control corresponding to each individual sample was utilized as a sample-matched reference baseline. The effect of each treatment was expressed as the fold change in mutation frequency (%) relative to the corresponding untreated control.

## 3 Results and discussion

### 3.1 Characteristics of test callus tissues used for quantification of mutation frequencies

CRISPR/Cas9 vectors are frequently based on *Agrobacterium*-mediated transformation systems, which generate stable transgenic plant cells constitutively expressing the Cas9 nuclease and guide RNA. In the present study, we utilized cultured callus tissues from two mutant lines to analyze the dependence of mutagenic efficiency on various culture conditions. Specifically, we selected two maize cell lines carrying knockout mutations in the *ARE1* and *KAT2* target genes. The integration of key genes from the CRISPR/Cas9 vectors was confirmed in the test cell lines (Figure 1).

**FIGURE 1:**
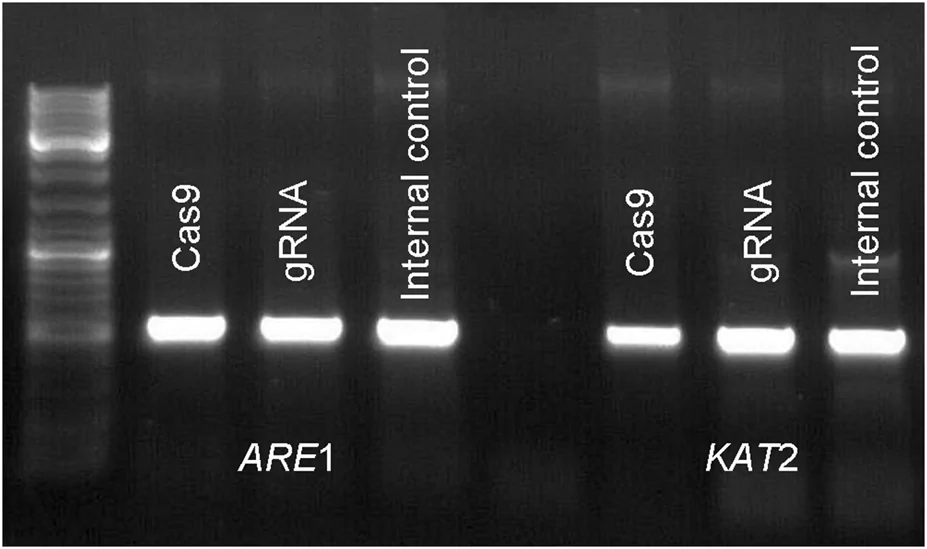
Agarose gel electrophoresis showing PCR amplification products from T-DNA from *ARE*1 and *KAT2* gene constructs in transgenic maize callus lines. Three primer sets were used for each construct: Cas9-specific primers (Cas9), gRNA-coding region-specific primers (gRNA), and internal control primers (internal control). The left panel shows *ARE*1 callus lines with positive amplification for Cas9, gRNA, and internal control, confirming successful integration of the CRISPR/Cas9 construct. The right panel shows *KAT2* callus lines with similar positive signals for all three primer sets (GeneRuler DNA Ladder Mix, #SM0331). The presence of amplification products in all tested samples confirms stable integration of the gene editing constructs in the callus tissues used.

Figure 2 illustrates the target sequence of the *ARE1* gene and the corresponding gRNA sequence, including the PAM motif. In the present CRISPR mutagenesis study of the *KAT2* gene, the transgenic callus tissues carried a 3′-truncated gRNA due to the deletion of an adenine (A) nucleotide immediately upstream of the PAM motif.

**FIGURE 2:**
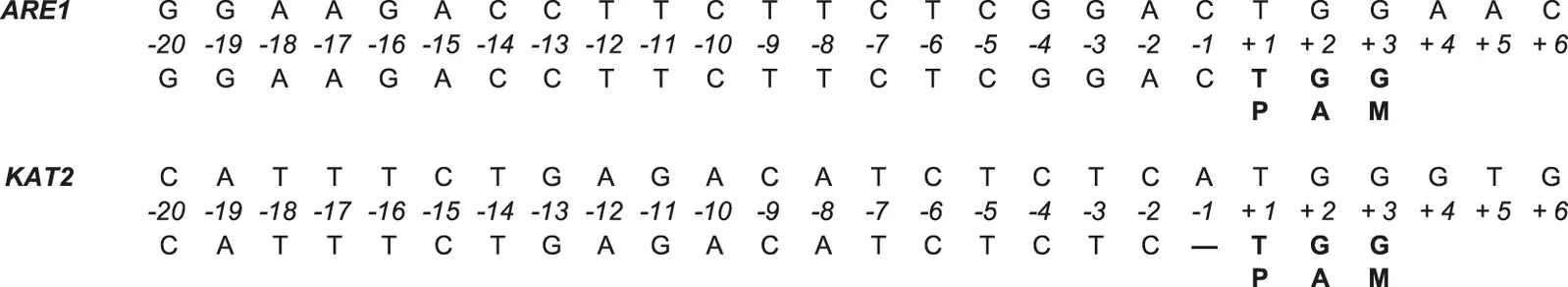
Nucleotide sequences of target genes and gRNAs in transgenic maize callus tissues. Nucleotide positions are numbered relative to the PAM sequence (bold letters). The *ARE*1 gRNA shows complete complementarity at all 20 positions. The *KAT*2 gRNA contains one nucleotide truncation at positions −1 (dash), immediately upstream of the PAM, resulting in missing complementarity at these positions.

Under standard culture conditions at 24 °C, the mutation frequency in the *KAT2* gene was approximately 100-fold lower than that observed in the *ARE1* gene (Table 1). Although the origin of this nucleotide deletion proximal to the PAM site remains unknown, the resulting truncation at the 3′end of the gRNA caused a substantial reduction in mutation frequency. Previously, Fu et al. (2014) reported that truncation at the distal 5′end of the gRNA could decrease undesired mutagenesis at off-target sites. Furthermore, target recognition was shown to be inhibited by single-base mismatches between the target DNA and 5′-truncated sgRNAs (Lee et al., 2021). However, these studies examined PAM-distal 5′truncations and therefore do not directly establish the functional consequences of the PAM-proximal 3′-end truncation observed in the *KAT2* gRNA. Our data about the cleavage of the *KAT2* gene by a gRNA lacking the −1 nucleotide can be interpreted as an indication for functionality of the truncated gRNA, but with essentially reduced efficiency. The observed improvement in editing efficiency under specific treatment conditions suggests that these treatments may modulate factors such as DNA accessibility, stability of the Cas9-DNA-gRNA complex, or activity of the DNA repair pathway, thus partially compensating for the reduced complementarity.

**TABLE 1:** The frequency of CRISPR/Cas9-induced indel mutations was preferentially elevated by the combined treatment of transgenic maize tissues with NaBt and heat at specific positions relative to the PAM site (*ARE1* deletion: -4 bp, insertion: -3 bp; *KAT2* deletion: -6 bp, insertion: -4 bp).

Target gene	Treatment	Mutation frequency %		
		Deletion	Insertion	InDEL
*ARE1*	Non-treated	3.350	3.000	6.350
	*(Total Counts)*	*1 553 498*	*1 609 760*	
	37 °C 48h	19.570	5.870	25.440
	*(Total Counts)*	*1 176 642*	*1 385 656*	
	Non-treated	2.930	1.270	4.200
	*(Total Counts)*	*335 078*	*345 911*	
	10 mM NaBt 48h	3.280	24.710	27.990
	*(Total Counts)*	*304 891*	*314 090*	
	Non-treated	2.930	1.270	4.200
	*(Total Counts)*	*335 078*	*345 911*	
	10 mM NaBt 48h → 37 °C 48h	24.170	21.940	46.110
	*(Total Counts)*	*201 329*	*252 426*	
*KAT2*	Non-treated	0.032	0.011	0.043
	*(Total Counts)*	*164 101*	*164 126*	
	37 °C 48h	0.206	0.068	0.274
	*(Total Counts)*	*427 904*	*428 379*	
	Non-treated	0.016	0.011	0.027
	*(Total Counts)*	*133 900*	*164 126*	
	10 mM NaBt 48h	0.090	0.000	0.090
	*(Total Counts)*	*133 900*	*133 900*	
	Non-treated	0.144	0.011	0.155
	*(Total Counts)*	*405 000*	*164 126*	
	10 mM NaBt 48h → 37 °C 48h	0.875	0.254	1.129
	*(Total Counts)*	*405 000*	*405 000*	

### 3.2 Modification of CRISPR/Cas9 mutagenic efficiency by heat, sodium butyrate (NaBt) treatment, and their combination at specific positions relative to the PAM site (*ARE1* gene: deletion at −4 bp/insertion at −3 bp; *KAT2* gene: deletion at −6 bp/insertion at −4 bp)

In the present study, we used maize callus tissues harboring components of the CRISPR/Cas9 system targeting two genes (*ARE1* and *KAT2*) to analyze the effects of heat, NaBt, and their combined treatments on the frequency of mutational events. Incubation of transgenic calli carrying the CRISPR/Cas9 machinery targeting the *ARE1* gene at an elevated temperature (37 °C for 48 h) increased the overall frequency of mutational events. This increase was particularly pronounced for nucleotide deletions (a 5.84-fold increase), whereas the insertion frequency increased only 1.96-fold (Table 1). In maize callus tissues carrying the CRISPR/Cas9 vector targeting the *KAT2* gene, the frequencies of both deletion and insertion mutations increased approximately sixfold.

Two primary factors can be considered when interpreting these effects. First, heat stress has been shown to alter DNA methylation and chromatin structure. For instance, Korotko et al. (2021) detected a reduction in DNA methylation levels several hours after subjecting *Arabidopsis* plants to heat stress (42 °C for 24 h), particularly in genes associated with the stress response. Because CpG methylation induces a closed and rigid nucleosomal conformation—resulting in a more tightly wrapped nucleosome structure (Choy et al., 2010) — a reduction in methylation leads to increased chromatin accessibility, as demonstrated by Zhong et al. (2021) in a profiling study of 18 different Arabidopsis DNA methylation mutants. The second factor is that the cleavage activity of SpCas9 is intrinsically higher at 37 °C compared to 22 °C (Kurokawa et al., 2021).

As shown in Table 1, NaBt, a known inhibitor of histone deacetylases (HDACs), increased the frequency of insertions nearly 19.5-fold, whereas the number of base-pair deletions was only slightly elevated in the *ARE1* gene. Previously, Lee et al. (2020) reported that NaBt significantly increased histone H3 acetylation in tobacco calli. Furthermore, pretreatment of maize cells with NaBt (5–10 mM) causes elevated chromatin sensitivity to DNase I, indicating a relaxed chromatin structure (Tiricz et al., 2018). The hyperacetylation of histones neutralizes their positive charges, loosening compact DNA and opening the chromatin. This increased chromatin accessibility allows the gRNA and Cas nucleases to reach target sites more effectively, significantly enhancing CRISPR cutting and editing efficiency (Björnson et al., 2023). Natural and engineered Cas9 variants can generate double-strand breaks with subtly different end configurations, ranging from blunt ends to occasional staggered ends carrying a 1-nt overhang. Because the chromatin structure is relaxed by HDAC inhibitors, endogenous DNA polymerases can immediately and easily access this single-base overhang. Prior to ligation, the polymerase rapidly fills the gap, with the nucleotide inserted depending on the local target sequence and break-end configuration. Because HDAC inhibition enables Cas9 to make significantly more cuts, and because polymerase-mediated end-filling prevails over nuclease-induced degradation (deletion) in most cases, the relative frequency of 1-bp insertions diverges substantially from that of deletions (Přibylová et al., 2022; Weiss et al., 2022). Interestingly, in the *KAT2* gene, treatment with 10 mM NaBt slightly decreased the number of insertion events while increasing the deletion frequency 5.7-fold.

A novel aspect of this study is the analysis of the combined effects of these two chromatin-modifying factors. In *ARE1* transformants, which already exhibited considerable mutagenic responses, the combined treatment produced only a moderate additive effect on nucleotide deletions relative to heat treatment alone, although this represented a substantial increase compared to the NaBt-only treatment. The frequency of nucleotide insertions following the combined treatment was slightly lower than that observed with NaBt alone. In contrast, in transformants exhibiting low basal CRISPR/Cas9 editing efficiency at the *KAT2* locus, a remarkable increase in both mutation types was recorded (a 6.1-fold increase in deletions and a 23.09-fold increase in insertions; Table 1). As discussed above, both heat and NaBt actively stimulate the frequency of mutational events, and their additive effect was particularly evident in overcoming the low editing efficiency at the *KAT2* target gene. Furthermore, during the combined NaBt and heat treatment, alongside the mechanisms leading to 1-bp insertions, the increased frequency and size of deleted fragments were consistent with a potential contribution of the MMEJ pathway (Figures 3A,B) (Yu and McVey, 2010; Wyatt et al., 2016; Schep et al., 2024).

**FIGURE 3:**
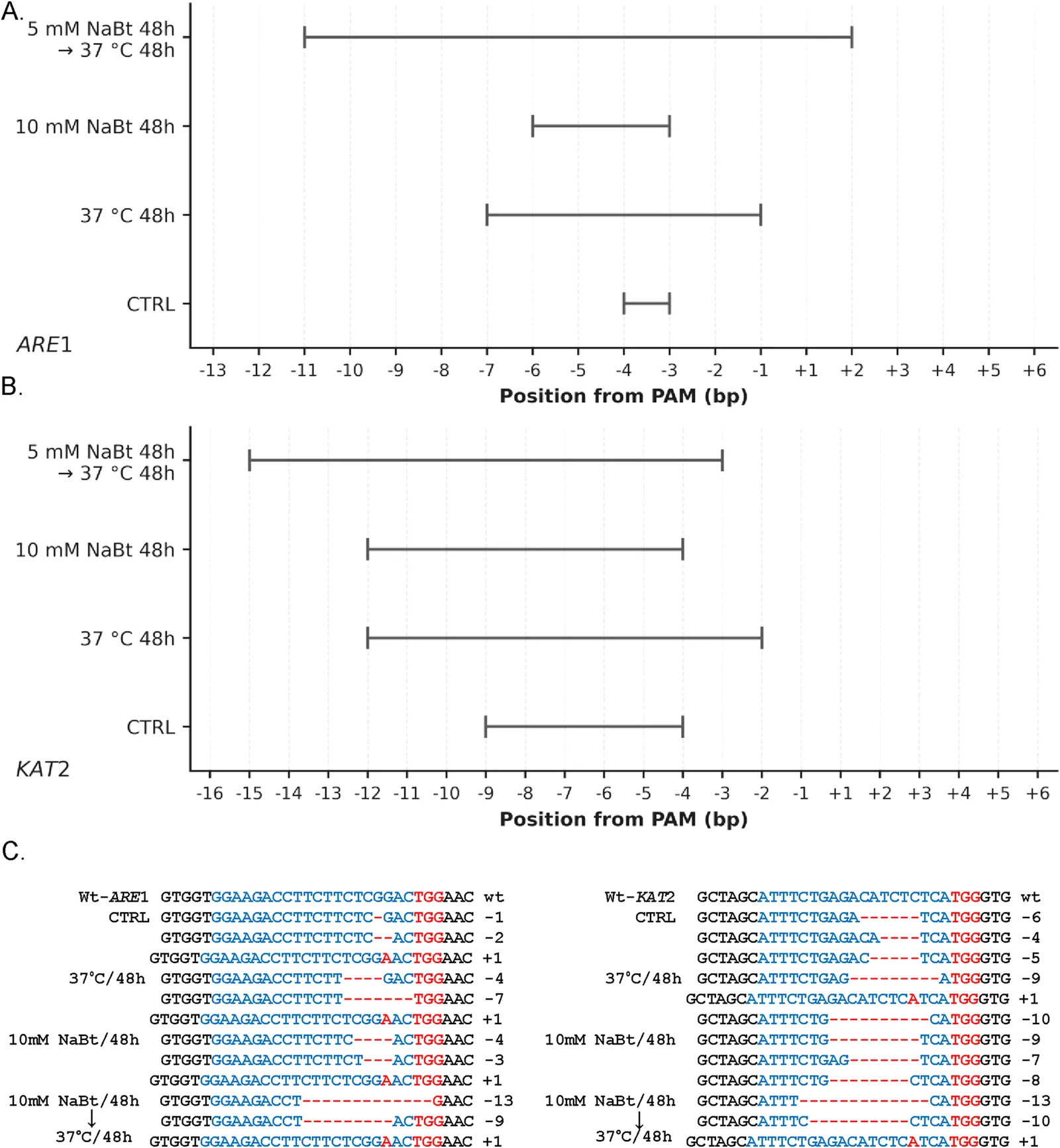
Positional distribution of nucleotide deletions relative to the PAM site. The combined treatment of NaBt and heat increased the number of deleted nucleotides in both test genes. The positions of the insertions were not affected by the different treatment combinations **(A)** The range and positions of deletions in the *ARE1* gene **(B)** The range and positions of deletions in the *KAT2* gene **(C)** Representative examples of the detected nucleotide changes.

DNA double-strand breaks (DSBs) generated by the Cas9 nuclease can be repaired via non-homologous end joining (NHEJ) or microhomology-mediated end joining (MMEJ). NHEJ-mediated repair events are predominantly utilized for gene knockouts, a process that is often only partially effective when considering the potential synthesis and residual function of the resulting protein product (Smits et al., 2019). Consequently, one can postulate that generating larger deletions can more reliably preclude the retention of target gene function. To investigate this, we monitored alterations in deletion sizes within the *ARE1* gene (Figures 3A,C).

In the untreated control transgenic CRISPR/Cas9 callus tissues, typically only one or two nucleotides were eliminated (Figures 3A,C). In heat-treated tissues, however, the number of eliminated nucleotides increased to a range of four to seven. NaBt treatment alone resulted in deletions of three to four nucleotides. Notably, the combined application of heat and NaBt produced a considerable increase in the size of the deleted fragments compared to either chromatin-modifying treatment alone. As illustrated in Figure 3B, the CRISPR vector utilizing a truncated gRNA for the *KAT2* gene induced more extended deletions even in the untreated control maize calli. The number of deleted nucleotides was further augmented following chromatin modification. As previously discussed, this phenomenon likely reflects an increased probability of DSB repair occurring via the MMEJ pathway.

### 3.3 Differential effects of nicotinamide (NA) and its combination with heat treatment on the frequency of nucleotide deletions and insertions in maize tissues targeting the *ARE1* or *KAT2* genes at specific positions relative to the PAM site (*ARE1* deletion: -4 bp/insertion: -3 bp; *KAT2* deletion: -6 bp/insertion: -4 bp)

NA also acts as a histone deacetylase inhibitor, causing elevated chromatin sensitivity to DNase I, which indicates a relaxed chromatin structure in cultured maize cells (Tiricz et al., 2018). As shown in Table 2, treatment of transgenic CRISPR/Cas9 callus tissues with 5 mM NA resulted in a 3.26-fold increase in the frequency of nucleotide deletions in the *ARE1* target gene. This was accompanied by the generation of large deletions (5–10 nucleotides), similar in size to those observed following the combined treatment (Figure 4A). Sequencing also revealed additive effects on the frequency of insertions.

**TABLE 2:** The frequency of CRISPR/Cas9-induced insertion mutations was preferentially elevated by the combined treatment of transgenic maize calli with NA and heat in the two targeted genes (*ARE1* and *KAT2*) at specific positions relative to the PAM site (*ARE1* deletion: -4 bp, insertion: -3 bp; *KAT2* deletion: -6 bp, insertion: -4 bp).

Target gene	Treatment	Mutation frequency %		
		Deletion	Insertion	InDEL
*ARE1*	Non-treated	3.350	3.000	6.350
	*(Total Counts)*	*1 553 498*	*1 609 760*	
	37 °C 48h	19.570	5.870	25.440
	*(Total Counts)*	*1 176 642*	*1 385 656*	
	Non-treated	6.340	2.850	9.190
	*(Total Counts)*	*672 395*	*715 196*	
	5 mM NA 48h	20.700	1.030	21.730
	*(Total Counts)*	*170 220*	*174 304*	
	Non-treated	6.340	2.850	9.190
	*(Total Counts)*	*672 395*	*715 196*	
	5 mM NA 48h → 37 °C 48h	14.390	14.670	29.060
	*(Total Counts)*	*254 000*	*286 367*	
*KAT2*	Non-treated	0.032	0.011	0.043
	*(Total Counts)*	*164 101*	*164 126*	
	37 °C 48h	0.206	0.068	0.274
	*(Total Counts)*	*427 904*	*428 379*	
	Non-treated	0.001	0.011	0.012
	*(Total Counts)*	*206 735*	*164 126*	
	5 mM NA 48h	0.019	0.000	0.019
	*(Total Counts)*	*206 700*	*206 700*	
	Non-treated	0.014	0.011	0.025
	*(Total Counts)*	*382 000*	*164 126*	
	5 mM NA 48h → 37 °C 48h	0.133	0.117	0.250
	*(Total Counts)*	*382 000*	*382 000*	

**FIGURE 4:**
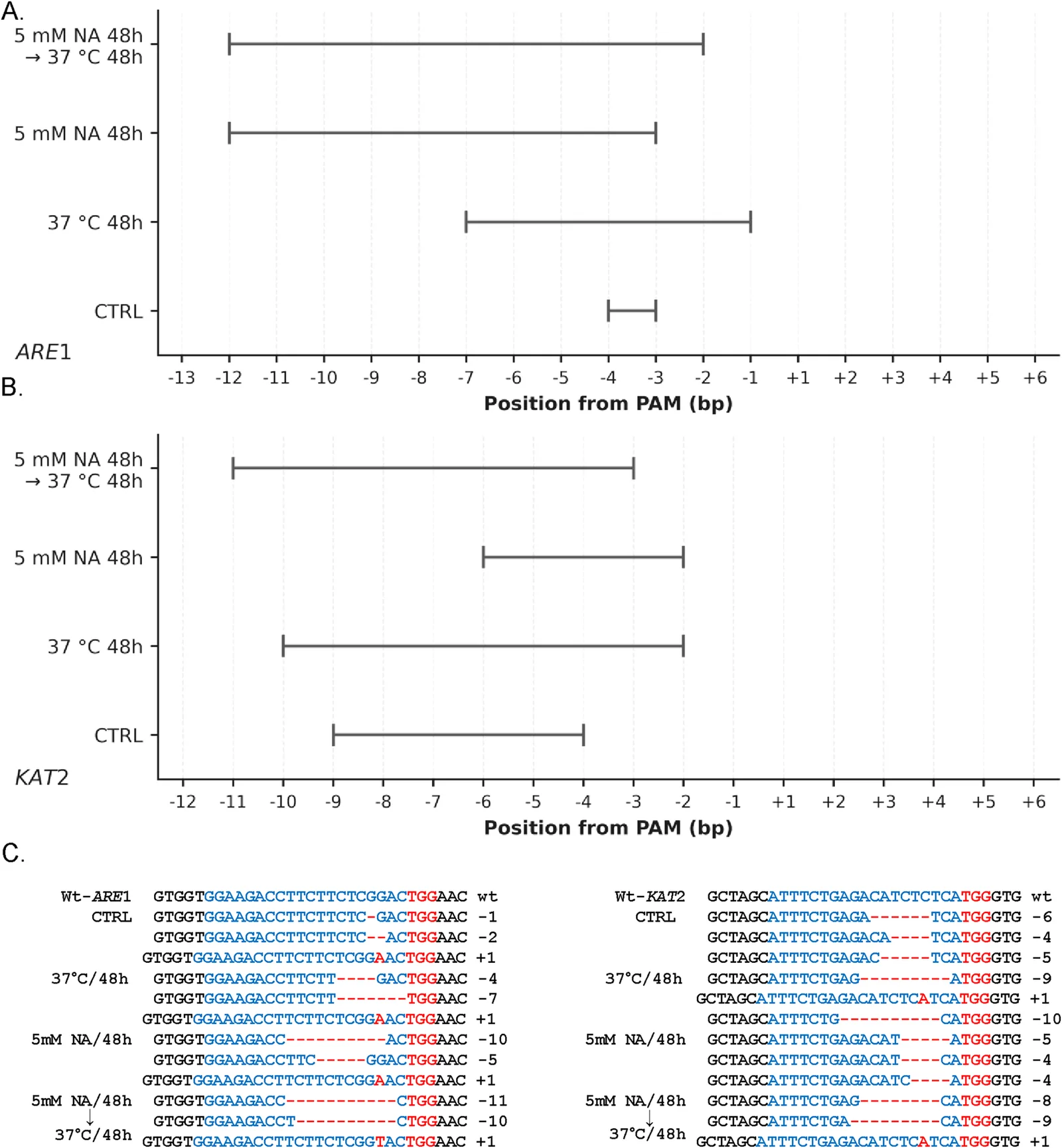
Positional distribution of deletions relative to the PAM site. NA treatment caused a marked change in the size of deletions within the *ARE1* test gene, and its combination with heat treatment further altered the profile of deletion sizes **(A)** The range and positions of deletions in the *ARE1* gene **(B)** The range and positions of deletions in the *KAT2* gene **(C)** Representative examples of the detected nucleotide changes.

In the *KAT2* target gene, CRISPR/Cas9-induced deletions were stimulated 19.4-fold following NA treatment. However, the length of the deleted regions was smaller than that observed in untreated control tissues (Figures 4B,C). The combined treatment preferentially increased the frequency of insertions compared to deletions. Consistently, Wang et al. (2023) reported that NA treatment of immature or mature wheat embryos increased the mutation frequency of the *GUS* and *TaWaxy* genes.

When comparing the NA results with those of NaBt—which is also an HDAC inhibitor—we observed that NA applied alone increased the deletion rate, whereas NaBt predominantly increased the insertion rate. When combined with heat, both NaBt and NA increased the rates of both deletions and insertions, although NA did so to a lesser extent. This discrepancy may be explained by the fact that NaBt is capable of opening up the chromatin structure to a greater degree, as we have previously demonstrated (Tiricz et al., 2018).

### 3.4 The frequency of CRISPR/Cas9-induced nucleotide insertion was preferentially elevated in *ARE*1 gene by 5-azacytidine (AZA), whereas the combined treatment of transgenic maize tissues with AZA and heat increased the number of both nucleotide insertions and deletions in the *KAT2* gene

As previously noted (Choy et al., 2010), CpG methylation induces a closed and rigid nucleosomal conformation, resulting on average in a more tightly wrapped structure. AZA is a small molecule analogous to cytidine that induces DNA hypomethylation by inhibiting the catalytic activity of DNA methyltransferases (Santi et al., 1983). Korotko et al. (2021) reported a reduction in DNA methylation levels several hours after subjecting *Arabidopsis* plants to heat stress (42 °C for 24 h). As presented in Table 3, treating CRISPR/Cas9 transgenic callus tissues targeting the *ARE1* gene with 10 µM AZA alone resulted in only a slight increase in nucleotide deletions, alongside a moderate increase in insertions. However, a substantial increase in nucleotide insertions was recorded following the combined treatment compared to either heat or AZA treatment alone. Interestingly, the rate of insertions increased more than that of deletions in response to AZA or the AZA-plus-heat treatment. When the chromatin structure of the *ARE1* locus was relaxed following treatment with the DNA methyltransferase inhibitor, cellular repair machinery efficiently filled in the “short overhangs” (staggered cuts) generated by Cas9, resulting in a predominance of short insertions (Přibylová et al., 2022). In contrast, when targeting the *KAT2* gene in CRISPR/Cas9 transgenic callus tissues, heat treatment caused an increase in both deletion and insertion events, whereas AZA treatment alone stimulated mutagenic activity by increasing the frequency of deletions 2.2-fold. The combination of heat and AZA treatments substantially elevated nucleotide modifications, leading to a 5.8-fold increase in deletions and a 21.36-fold increase in insertions (Table 3). The observed differences between the two targeted genes can be explained by genome-wide methylome analyses demonstrating that demethylation induced by AZA can be highly locus- or gene-specific (Xu et al., 2017).

**TABLE 3:** The frequency of CRISPR/Cas9-induced indel mutations was preferentially elevated by the combined treatment of transgenic maize calli with AZA and heat at specific positions relative to the PAM site (*ARE1* deletion: -4 bp, insertion: -3 bp; *KAT2* deletion: -6 bp, insertion: -4 bp).

Target gene	Treatment	Mutation frequency %		
		Deletion	Insertion	InDEL
*ARE1*	Non-treated	3.350	3.000	6.350
	*(Total Counts)*	*1 553 498*	*1 609 760*	
	37 °C 48h	19.570	5.870	25.440
	*(Total Counts)*	*1 176 642*	*1 385 656*	
	Non-treated	4.300	1.670	5.970
	*(Total Counts)*	*248 937*	*257 679*	
	10 µM AZA 72h	4.860	4.520	9.380
	*(Total Counts)*	*256 878*	*262 719*	
	Non-treated	8.960	1.400	10.360
	*(Total Counts)*	*588 488*	*643 944*	
	10 µM AZA 72h → 37 °C 48h	13.680	16.950	30.630
	*(Total Counts)*	257 889	*290 927*	
*KAT2*	Non-treated	0.032	0.011	0.043
	*(Total Counts)*	*164 101*	*164 126*	
	37 °C 48h	0.206	0.068	0.274
	*(Total Counts)*	*427 904*	*428 379*	
	Non-treated	0.130	0.011	0.141
	*(Total Counts)*	*381 000*	*164 126*	
	10 µM AZA 72h	0.280	0.005	0.285
	*(Total Counts)*	*381 000*	*216 900*	
	Non-treated	0.077	0.011	0.088
	*(Total Counts)*	*463 000*	*164 126*	
	10 µM AZA 72h → 37 °C 48h	0.450	0.235	0.685
	*(Total Counts)*	*463 000*	*463 000*	

Sequence analysis of the *ARE1* gene in AZA-treated transgenic calli revealed that this chromatin-modifying compound did not increase the size of the deleted regions. However, the combination of heat and AZA treatments resulted in deletions spanning 8 to 10 nucleotides (Figure 5). In the *KAT2* gene, both heat and AZA treatments increased mutation efficiency, demonstrating a clear additive effect.

**FIGURE 5:**
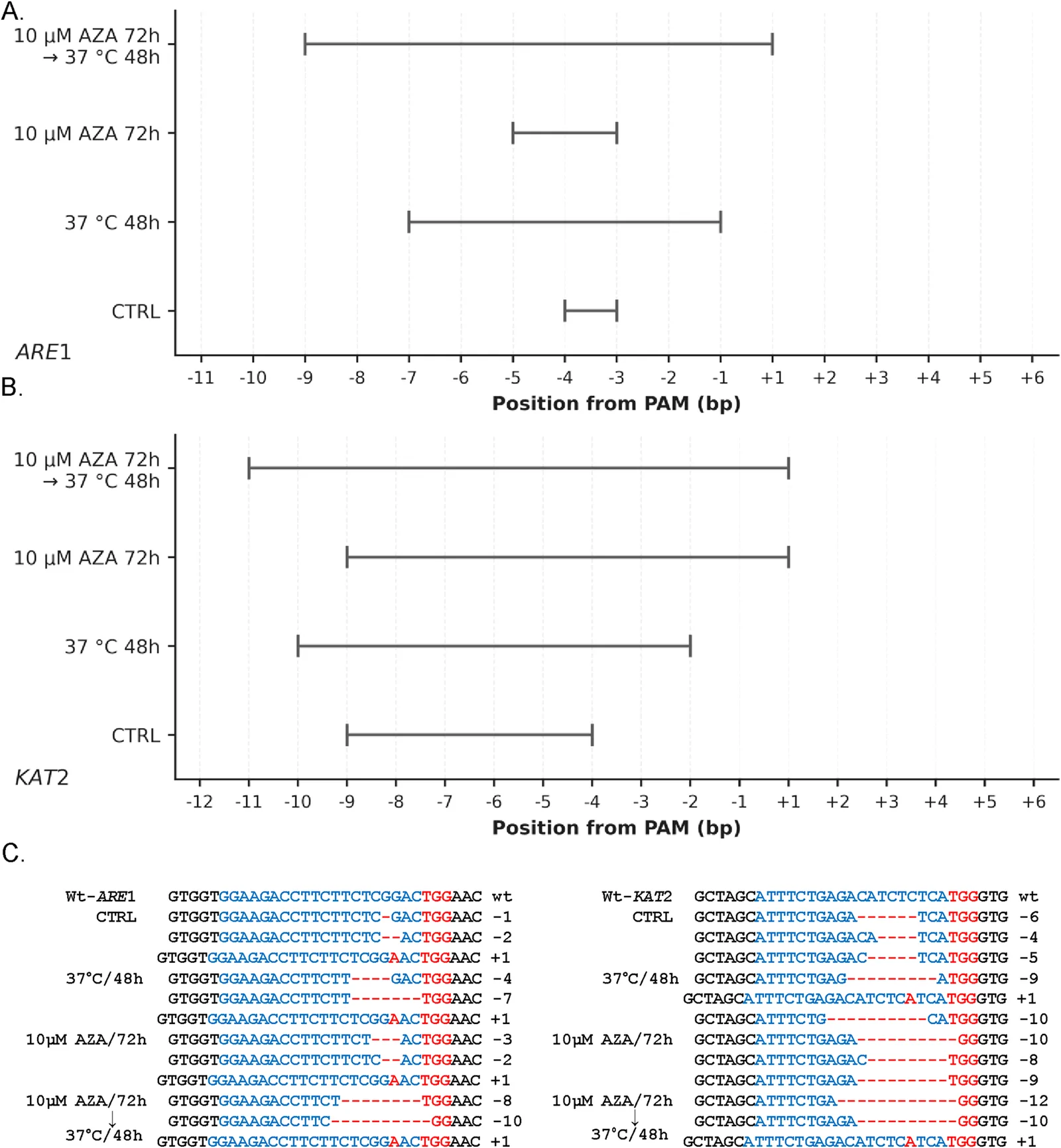
Positional distribution of deletions and insertions relative to the PAM site. The combined treatment of AZA and heat substantially increased the number of deleted nucleotides in the *ARE1* test gene. The size of the deletions in the *KAT2* gene increased following heat, AZA, and the combined treatment of the CRISPR/Cas9 transgenic maize calli. The positions of the insertions were not affected by the different treatment combinations **(A)** The range and positions of deletions in the *ARE1* gene **(B)** The range and positions of deletions in the *KAT2* gene **(C)** Representative examples of the detected nucleotide changes.

### 3.5 Chromatin-modifying compounds, including heat treatment, alter the cell cycle phase distribution in CRISPR/Cas9 transgenic maize suspension cells

The preceding data clearly indicate alterations in the frequency of deletions and insertions following the various applied treatments. Both mutagenic events are inherently dependent on the specific DNA repair mechanisms employed, and the activity of these repair pathways fluctuates throughout cell cycle progression. Major DNA double-strand break repair pathways include non-homologous end joining (NHEJ) and microhomology-mediated end joining (MMEJ). Whereas NHEJ is active throughout all cell cycle stages and involves the direct ligation of broken DNA ends—enabling fast repair, albeit with the potential for introducing errors—MMEJ activity ensures that cells do not commit to cellular division with unrepaired breaks originating from the S-phase (reviewed by Sfeir et al. (2024); Přibylová and Fischer (2024).

To gain insight into how the applied treatments alter cell cycle progression, we utilized fast-growing maize suspension culture cells as a model system. Because callus cultures are suboptimal for nuclei isolation and flow cytometry, the subsequent experiments were performed on cell suspensions.

As illustrated in Figure 6, the majority of cells in untreated control cultures resided in the G0/G1 phase (∼65%), with smaller fractions in the S (∼18%) and G2 (∼17%) phases, consistent with the normal distribution of an asynchronously proliferating population. Heat stress alone led to an accumulation of cells in the S and G2 phases—indicative of replication stress and activation of the G2/M checkpoint—alongside a concomitant, significant decrease in the G0/G1 fraction (p < 0.05). This physiological shift is consistent with previous studies demonstrating that abiotic stresses, such as heat, induce dynamic changes in the regulation of cell cycle genes and checkpoints in plants (Jang et al., 2005; Zhao et al., 2014). Furthermore, the application of chemical modulators (NA, NaBt, and AZA) in combination with heat (H + NA, H + NaBt, and H + AZA) provoked profound and statistically significant redistributions across all phases of the cell cycle. Both the H + NA and H + NaBt treatments similarly reduced the G1 population to approximately 44%–46% while driving a significant accumulation of cells into the G2 phase (∼30%). In particular, the combined treatment of heat and NaBt (H + NaBt) proved to be the most potent combination for reducing the number of mitotic cells (Figure 7). As detailed in Tables 1–3, the combined heat and chemical treatments produced the highest frequency of nucleotide deletions and insertions in the *ARE1* gene. Interestingly, the combined effect of heat and AZA caused a more pronounced alteration in the DNA synthesis and mitotic phases (Figures 6, 7).

**FIGURE 6:**
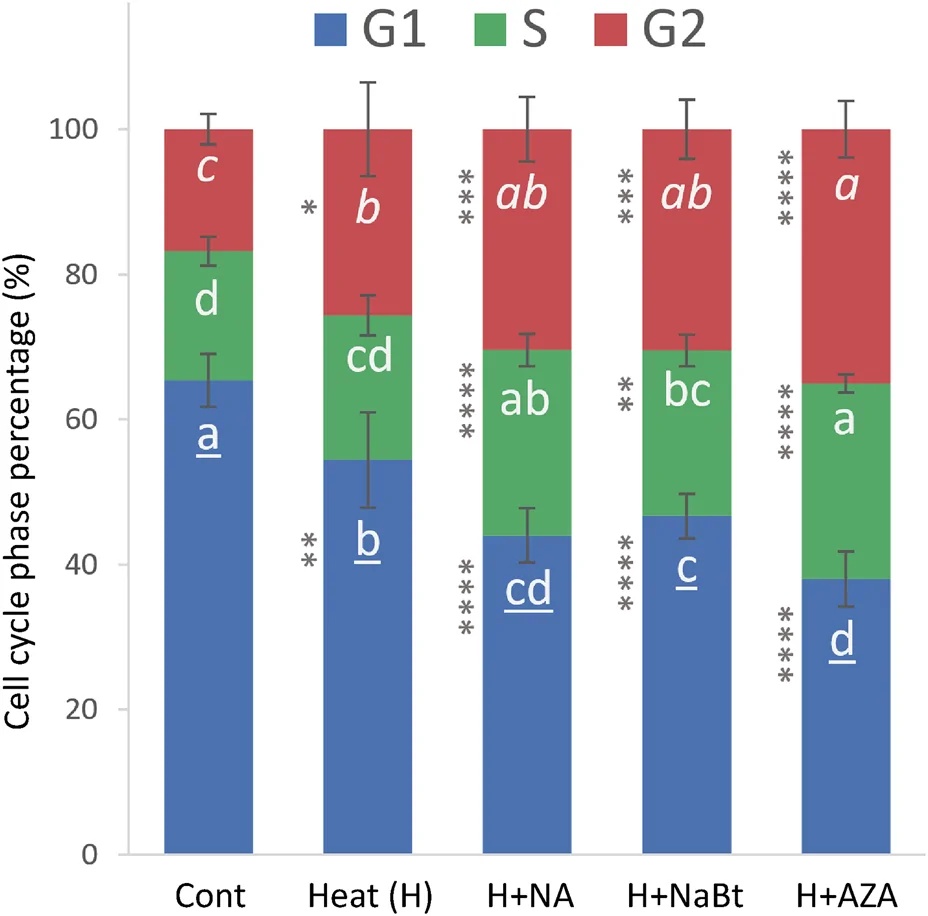
The effect of combined heat and inhibitor treatments on cell division cycle phase distribution. The distribution of the G1, S, G2 phases is expressed as the mean ± standard deviation (n = 6 repetitions from 2 independently subcultured flasks). Different letters indicate significant differences between groups based on one-way analysis of variance (ANOVA) followed by Tukey’s multiple comparison test (p < 0.05). The asterisks indicate levels of statistical significance compared to the control: *p < 0.05, **p < 0.01, ***p < 0.001, ****p < 0.0001. H: heat treatment (37 °C), NA: nicotinamide, NaBt: sodium butyrate, AZA: 5-azacytidine.

**FIGURE 7:**
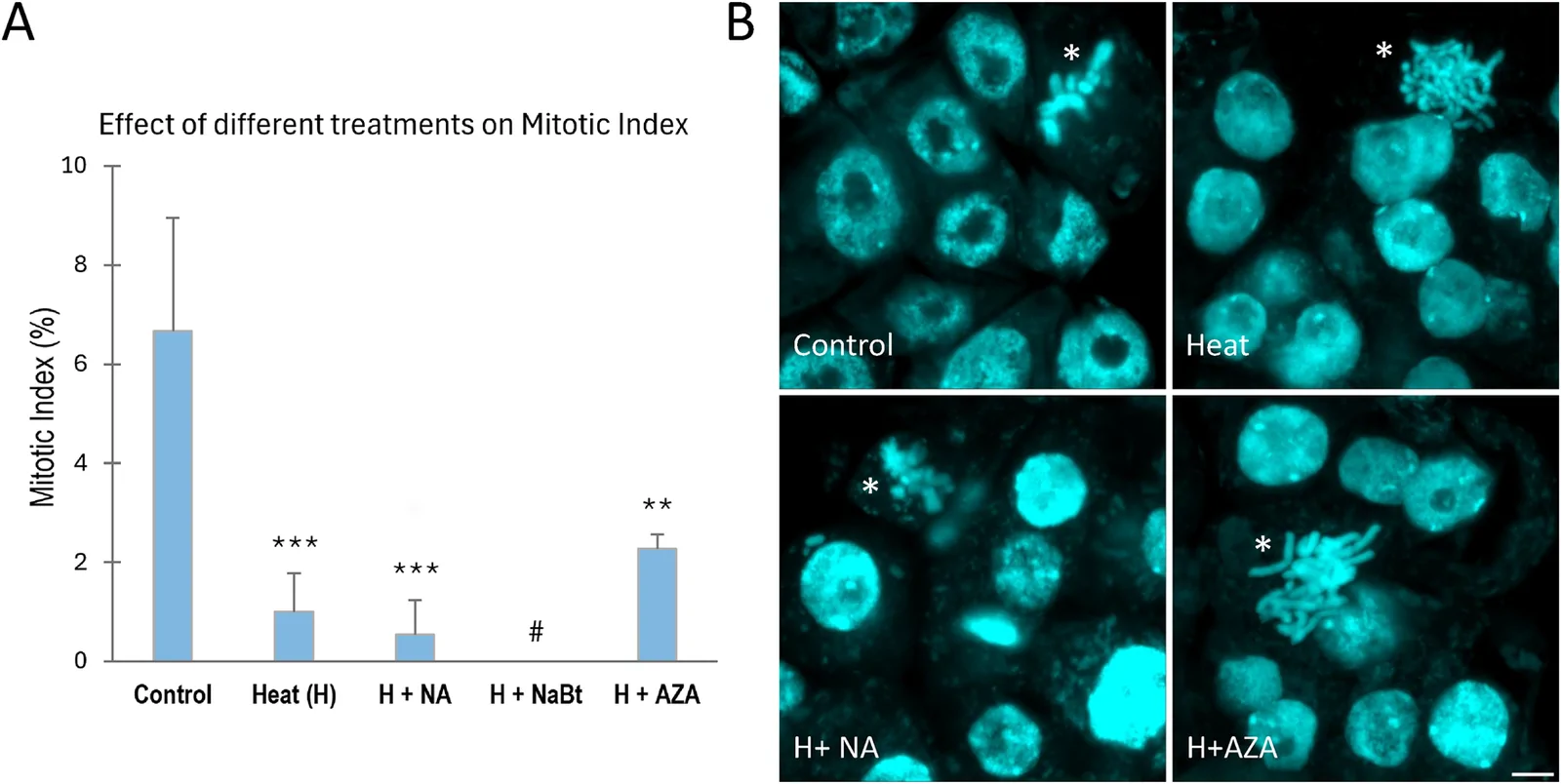
**(A)** The effect of heat and combined inhibitor treatments on the mitotic index (%) of maize cells. The bars show mean ± SD of 3 replicates. Statistical analysis was performed using a one-way analysis of variance (ANOVA) followed by Tukey’s *post hoc* test for multiple comparisons. Asterisks indicate the levels of statistically significant differences between the groups as compared to the control (**p < 0.01, ***p < 0.001). #, no mitotic figures were observed in the H + NaBt sample **(B)** Confocal microscopy images of representative mitotic cells. The scale bar is 5 µm. Asterisks indicate condensation of mitotic chromosomes. H: heat, NA: nicotinamide, NaBt: sodium butyrate, AZA: 5-azacytidine.

## 4 Conclusions

The outcome of gene-specific editing depends on both the gene-editing constructs and the genomic architecture of the recipient cells. The presented test system, based on transgenic maize callus tissues carrying a CRISPR/Cas9 vector targeting either the *abnormal cytokinin response 1* (*ARE1*) or the *potassium channel* (*KAT2*) gene, allowed us to demonstrate *via* DNA sequencing that both nucleotide deletions and insertions can be generated following a prolonged culture period (15 months). Notably, the *KAT2*-targeting vector with a 3′-end truncated guide RNA (−1 nucleotide upstream of the PAM site) also exhibited mutagenic activity, albeit resulting in a very low frequency of indels.

Culturing the test callus tissues at 37 °C for 48 h preferentially increased the frequency of nucleotide deletions in the *ARE1* target gene, whereas insertions and deletions occurred at similar frequencies in the *KAT2* locus.

Treatment with any of the three chromatin-modifying compounds alone (with the exception of NaBt in the *ARE1* gene) resulted in a smaller increase in gene-editing efficiency compared to heat treatment alone or combined treatments. For both genes, combined treatments produced a greater increase in insertion events than in deletions. Specifically, the combined application of NaBt and heat for *ARE1*, and NA and heat for *KAT2*, increased the frequency of the indels to the highest values observed in the present experiments. Furthermore, in the *KAT2* gene, all combined treatments proved more efficient in increasing indel percentages than either heat or chemical treatment alone. Based on the literature, we postulate that the optimal function of the Cas9 nuclease at elevated temperatures, alterations in chromatin structure, the nature of the Cas9 cleavage (blunt-end or staggered DNA cut), the balance of post-CRISPR repair pathways, and the potential involvement of the MMEJ pathway could collectively account for the observed increase in mutagenic response.

Among the three chromatin-modifying compounds, NaBt alone was the most effective in increasing insertion frequency in the *ARE1* gene, while NaBt combined with heat induced the highest number of deletions. For the *KAT2* gene, the most substantial increase in insertions was observed following the NaBt and heat combination, whereas the highest increase in deletions occurred with NA alone. The additive effects between heat treatment and various chromatin structure modifiers appear to be a possible trend, albeit with variable efficiency. A substantial increase in the frequency of both deletions and insertions was generated by the combination of heat and NaBt across both test genes. Combining heat treatment with AZA or NA enhanced deletion and insertion mutagenesis in both the *ARE1* and *KAT2* genes. Additionally, the sequencing data revealed an expansion in the size of the deleted regions within the mutated genes.

To gain insight into the mechanisms underlying these mutagenic events, we characterized alterations in cell cycle phase distribution. All treatment combinations reduced the proportion of mitotic cells while increasing the number of cells in the S and G2 phases. These findings suggest that a novel methodology combining heat treatment with chromatin-modifying compounds can be utilized for improved gene editing in plant cells.

Finally, we must acknowledge certain limitations in interpreting these results. This test system relies on dedifferentiated callus cells, which possess altered cell division and chromatin characteristics compared to intact plant organ cells. However, because CRISPR/Cas9-based editing protocols generally involve a callus stage prior to plant regeneration, this experimental setup provides a biologically relevant model for evaluating factors that influence editing efficiency. Due to sample pooling as a technical limitation, biological replicates were not available for further statistical analysis. Additionally, we note that the IGV-based workflow reports only the predominant variant at the position of maximum frequency, rather than providing a comprehensive size-frequency distribution. Finally, we acknowledge that the present study is restricted to only two maize genes.

## Data Availability

The datasets presented in this study can be found in online repository. The name of the repository and accession number can be found below: Mendeley Data, doi: 10.17632/fm4h3mphcr.1. The dataset includes (i) NGS raw data (ii) Fig7B Microscopy Originals.lif. (iii) @Fig6 FACS original data.zip.
